# Supplementary material for: Multimodal imaging and functional analysis of the chick NMDA retinal damage model
Source: PLoS One. 2021 Sep 7;16(9):e0257148. doi: 10.1371/journal.pone.0257148 (PMC8423281; doi:10.1371/journal.pone.0257148)
Supplement: S1 File — (ZIP) [file pone.0257148.s004.zip › SD-OCT/C200727/017/D28-Pwk5/C200727--017_OS_V_14X14_0_REGAVG0000097/Manual_PreDefineFiveByFiveGridRectangularRetina_1.pdf]

Five By Five Grid Template Report

Patient ID	C200727	Name	See InVivoVue
Exam Date	See InVivoVue	Exam Time	See InVivoVue
Eye	OS	Averaged	N/A
Depth (mm)	1.65	Depth Samples	1024
Pitch (um/pix)	35.000 x 35.000 x 1.608	Refractive Index	See InVivoVue
Scan Type	Rectangular 14.000mm x 14.000mm x 1.647mm , 400 x 400 x 1024		

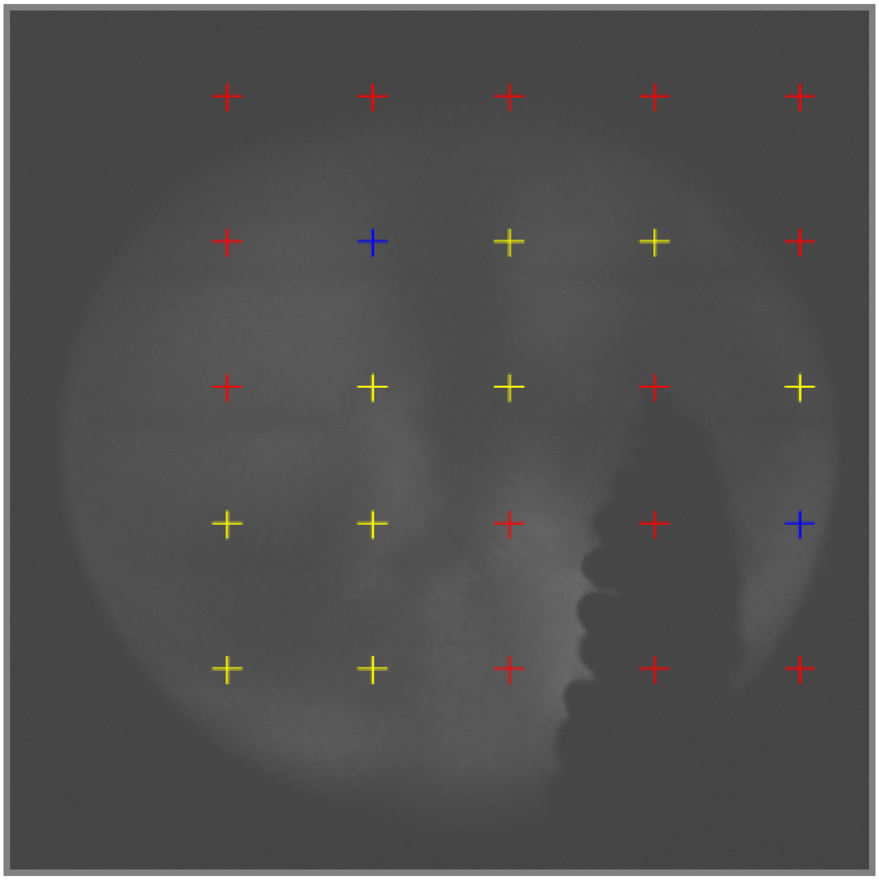

Thickness Metrics

Analysis	Average(mm)	Minimum(mm)	Maximum(mm)	STDEV(mm)
IRNFL - RPE	0.2612	0.2428	0.2796	0.0260
IRNFL - ORNFL	0.0431	0.0250	0.0612	0.0256
ORNFL - OPL	0.1389	0.1086	0.1691	0.0428
OPL - ETPRS	0.0596	0.0543	0.0649	0.0075
